# Supplementary material for: Melatonin Modulates the Antioxidant Defenses and the Expression of Proinflammatory Mediators in Pancreatic Stellate Cells Subjected to Hypoxia
Source: Antioxidants (Basel). 2021 Apr 8;10(4):577. doi: 10.3390/antiox10040577 (PMC8070371; doi:10.3390/antiox10040577)

Supplementary figure 1. Detection of specific markers for activated PSC by confocal microscopy. The method that we employed for the obtention of primary cultures of rat PSC allows the obtention of cells in an activated state. The conditions of isolation and culture are optimized for the obtention and growth of viable PSC. The purity of the preparations was tested. For this purpose, cells were stained with antibodies for specific markers of PSCs as α-smooth muscle actin (A) or collagen type 1 (B). Nuclei were stained with DAPI. All fields analysed were positive stained for these markers, therefore confirming that PSC in culture are cells in an activated state. Dapi and the primary antibody against α-smooth muscle actin were purchased from Fisher Scientific Inc. (Madrid, Spain). The primary antibody against collagen type 1 was obtained from Abcam (Abcam plc, Cambridge, UK). The corresponding fluorescent-conjugated secondary antibody (green/red) was purchased from Fisher Scientific Inc. (Madrid, Spain). The white bar length corresponds to 50 μm.

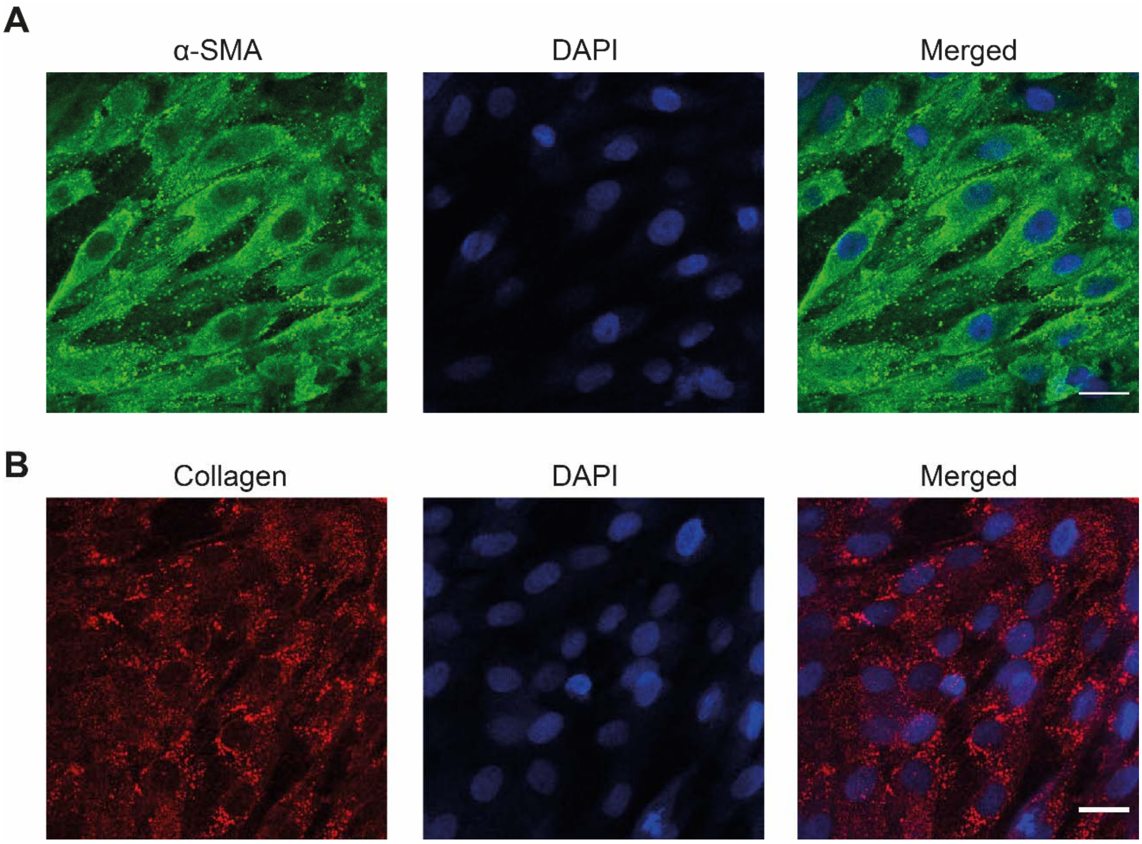

Supplement: Supplementary file 1 [file antioxidants-10-00577-s001.pdf]
